# Religiosity of Migrants and Natives in Western Europe 2002–2018: Convergence and Divergence

**DOI:** 10.1007/s10680-023-09660-9

**Published:** 2023-03-23

**Authors:** Ayse Guveli, Lucinda Platt

**Affiliations:** 1 Department of Sociology, University of Essex, Wivenhoe Park, Essex, Colchester, CO4 3SQ UK; 2 Department of Social Policy, London School of Economics and Political Science, Houghton Street, London, WC2A 2AE UK

**Keywords:** Religiosity, Migrants, Second generation, Europe, Trends, Secularization

## Abstract

Patterns of religiosity among both settled and migrant populations have been the subject of intense, and often conflicting, scholarly debate. In Europe, most analysis of migrant religiosity has focused on Islam, though migrants to Western European countries come from a wide range of religions and denominations. Despite a general assumption of assimilation over generations to greater secularization, evidence on trends in religiosity across migrants of different religions and for both first and second generations remains partial. We use the European Social Survey (rounds 1–9) to examine three dimensions of religiosity encompassing both performative and subjective domains, across 15 Western European destination countries over a 16-year period. While variation in religiosity between different affiliations is not large, migrants tend to have higher religiosity than non-migrants across the religious affiliations we consider. Over time we see that while natives show an overall decline in religiosity over the period, first- and second-generation Protestants and Muslims show increases in religiosity, providing some evidence for religious revival. We discuss the implications of our findings for theories of secularization and religious revival, and the future religious landscape of Europe.

**Supplementary Information:**

The online version contains supplementary material available at 10.1007/s10680-023-09660-9.

## Introduction

The religious involvement of migrants and their descendants has the potential to reshape the religious landscape of Europe. Migrants come with religious affiliations that often differ from those historically dominant in destination countries. In addition, those of migrant origin may also show greater religiosity in their given religion, not only in the first (Alecksynska & Chiswick 2013), but potentially also in the second generation (De Hoon & van Tubergen, 2014). They may therefore contribute to slowing down general processes of secularization in religious expression. How far countries are changing in their religious profile, and the extent to which migration is contributing to that, is a salient issue, with little concrete evidence.

Understanding the patterns and trends in migrant religiosity across religious affiliations is thus of substantial interest. This is especially the case given, on the one hand, the transformations in immigration to and within Europe over recent decades (Van Mol & de Valk 2016), and on the other, the increasing degree of organization and community-forming of the second generation of earlier migration flows (Alba & Foner, 2015). Yet, despite a growing body of research on migrant religiosity across Europe, we have little insight into how far religiosity differs between migrants, the second generation and non-migrants of different religions. Despite the wealth of attention to Muslim religiosity, we do not know how far Islam is exceptional. We additionally lack understanding of whether the religiosity of migrants, second generation, and non-migrants, of different religions and none, is converging or diverging both across generations and over recent years. These are the concerns of this paper.

Most European countries have experienced substantial migration, both from within Europe and from former colonial countries, as well as from countries such as Turkey and Morocco, which supplied labour migrants in large numbers to many Western European countries. More recently, the collapse of the former Soviet bloc and wars in the Balkans, Afghanistan and the Middle East generated new migration flows (Castles, et al., 2003; Pollack & Rosta, 2017). The ‘newer’ immigration countries of Italy, Portugal and Spain now have substantial shares of migrants, many coming from Latin America, and a growing second generation (Cebolla-Boado & Finotelli, 2015). The EU and its enlargement in 2004 and 2007 also increased the movement of Europeans with different levels of religiosity to countries other than those of their birth (Koenig et al., 2016). Those of migrant origin, from a diverse set of religious denominations, now represent a significant share of the population of many European countries. This share is set to grow through natural increase even without further migration (Pew Research Center, 2018a). As this happens, migrants’ religiosity and the extent to which it is effectively transmitted to subsequent generations (Ebaugh & Chafetz, 2000) will shape overall religious commitment and patterns of secularization in Western Europe (Spohn, 2009).

Migrants’ religiosity has witnessed increased attention from researchers in recent years. However, the primary focus in European research, driven extensively by wider concerns with cultural difference and threat (Parekh, 2006), has been on Muslim religiosity (e.g. Fleischmann & Phalet, 2012; Voas & Fleischmann, 2012; Maliepaard et al., 2010b; Guveli & Platt, 2011; Guveli, 2015; Connor, 2010). These studies cannot, by definition, reveal how far Muslims differ from or are similar to other migrant religious groups (cf. Voas & Fleischmann, 2012). As noted by Alba and Foner (2008), the emphasis on Muslim religiosity as a source of anxiety differs from that in the US literature where religion is seen as a means of inclusion or integration. This focus on Muslim religious expression can therefore hinder a more balanced and comprehensive understanding of patterns of migrant religiosity. In addition, given the ways in which Christian religiosity is evolving in the West, with recent findings of greater religious expression among Protestants (Wilkins-Laflamme, 2016), it is relevant to understand how far migrants are part of such changes in religiosity in Christian denominations (cf. also Molteni & van Tubergen, 2022).

We are missing multi-country studies of whether migrants and the second generation from multiple religions are more religious than natives. We also do not know whether migrant religiosity is declining or stable in recent years. In existing cross-national and cross-sectional studies of migrant religiosity, the focus has primarily been on the migrant generation, either with (e.g. Aleksynska Chiswick, 2013; Guveli, 2015) or without (e.g. van Tubergen, 2006; van Tubergen & Sindradóttir, 2011) further comparison with the native majority. This is an important gap, given that the second generation is arguably a better indicator of the long-term trends in religiosity. Migrant religiosity is influenced by compositional differences in flows, in response both to immigration policy and world affairs, by changing patterns of selection (Feliciano, 2020), and by differences in the religiosity of return migrants, alongside any adaptation that takes place in the destination country context. This is not the case for the second generation.

Finally, religiosity cannot effectively be captured with a single measure. Different dimensions of religiosity are substantively informative about religious maintenance or decline. They are differently linked to religious affiliations. For example, attendance is more institutionalized in some religions than in others, and private prayer is more typical in others. Compared to private prayer, attendance at places of religious worship requires the availability of institutional settings, but may also provide additional resources that benefit migrants in a challenging new context (Breton, 1964; Phillips et al., 2007). Again, while these dimensions have been explored for Muslim migrants (Guveli, 2015), there is limited work that compares different dimensions across migrants of different religions—or between the first and second generation. In sum, our study addresses the following questions:

1. To what extent do first-generation migrants, the second generation and natives differ in their religious commitment in European countries, and how does this vary across religions and across dimensions of religiosity?
2. To what extent does the religiosity of first-generation migrants and the second generation converge with or diverge from native populations across different religious groups over the period of our study?

Existing literature provides little precise guidance as to what we might expect the answers to these questions to be. Our analysis is therefore primarily exploratory, and intended to establish findings on contemporary patterns of religiosity and secularism that can inform further work in this area. To address these questions, we analyse nine rounds of the European Social Survey (ESS) 2002–2018, a study that has been used extensively for research on those of migrant origin, for the study of religiosity, and for comparisons with non-migrants (e.g. Guveli, 2015; Aleksynska & Chiswick 2013; Guveli & Spierings, 2021; Molteni & van Tubergen, 2022). We compare three dimensions of religiosity—praying, attendance at a place of religious worship, and subjective religiosity–across different religions for natives, migrants and the second generation. We then examine trends over the period 2002–2018. We estimate models with destination country fixed effects and adjust for individual characteristics associated with religiosity and that differ between migrants, the second generation and natives.

## Background and Previous Findings

To motivate our study and provide relevant context for our findings, we outline existing research on (migrant) religiosity in Europe.

European societies are secularizing (Bruce, 2011). Despite claims about different forms of religious expression or spirituality (Davie, 2000; Norris & Inglehart, 2011; Woodhead, 2009), the weight of evidence indicates a steady decline in both ‘believing and belonging’ (Bruce, 2011; Smith & Kim, 2005; Voas & Crockett, 2005) even if it has begun later in different parts of the continent (Wilkins-Laflamme, 2016). Wilkins-Laflamme (2016) has also identified heightened levels of religious expression among the declining share of Protestants in Western societies, which points to retention or even increase in religiosity among those who remain affiliated. Patterns of *religiosity* among the majority population thus do not necessarily reflect patterns of affiliation across denominations.

Religion is increasingly chosen rather than ascribed. According to recent research, people increasingly pick and choose their own form of piety (Davie et al., 2017). On the one hand, some people continue to affiliate to the religion they have inherited from their parents, even if not practising it. Others do not affiliate to any institutionalized religion but still demonstrate some attention to rituals and spirituality. For example, non-affiliated may pray to God or a ‘higher power’ or subjectively consider themselves religious in their ‘personal’ beliefs. This points to the importance of investigating beliefs and practices alongside affiliation to better understand the extent and patterns of religiosity in Europe.

### Domains of Religiosity

Religion or religious attachment can be represented in different ways. Those who contest the tenets of secularization theory (e.g. Davie, 1994; Davie et al., 2017) have emphasized the importance of individualization of religious practice relative to institutional behaviours. While highly correlated in general, each dimension of religiosity may play out differently by migration status and context.

Affiliation to a religion indicates the cultural and historical totality of the person’s belonging since religion and culture are often interlinked (Davie, 1994). Religious observance, by contrast, demonstrates commitment to the practical side of faith. It can itself be separated into communal forms (such as attendance at places of worship) and personal forms (such as private prayer). Furthermore, the subjective importance of religion represents a further way in which people may assert their spirituality, without necessarily implying any behavioural connotations (Davie, 1994). Secularization arguably impacts behavioural forms of religiosity more than adherence to spirituality, leading to declines in attendance (and prayer) that are not necessarily matched by declines in adherence to some form of faith and the felt importance of that faith.

But the relative patterns of attendance, prayer and subjective religiosity may also differ for migrants. Migrants are likely to benefit from institutional forms of religion for the resources and support offered, and the ways they help individuals to adjust to Western European secular societies and reformulate their religion and identity (cf. Guveli, 2015). Individual forms of religiosity which people observe in their personal domains such as prayer, by contrast, receive less positive reinforcement and are more subject to secularizing forces, particularly among the second generation. To the extent that subjective religiosity is interlinked with identity, as that identity is made salient in a foreign context or becomes an alternative to ethnic belonging for the second generation, it may better resist secularizing trends (Drouhot, 2021; Guveli & Platt, 2011; Jacobson, 1997; Platt, 2014, Molteni & van Tubergen, 2022).

### Religion and Migration in European Societies

European societies are affected by increasing migration. There has been extensive academic interest in recent years in the consequences of increasing international migration for the religious landscape and structure of the destination societies (e.g. Levitt, 2007; Hagan & Ebaugh, 2003; Smith & Kim, 2005; Wuthnow & Offutt, 2008; Yang & Ebaugh, 2001; Voas & Fleischmann, 2012; Maliepaard et al., 2010b; Guveli et al., 2016a; Guveli & Platt, 2011). As migration increases, so does the diversity of religions. Europe has experienced population movement from different regions, and from countries with a wide range of dominant religions. Given the more youthful profile of migrants, accompanied in some cases by higher fertility (Kulu & González-Ferrer, 2014), increases in religious diversity are likely to continue through demographic processes even in the absence of further migration (e.g. Pew Research Centre 2017). There is also some evidence that migrants are more effective in transmitting their religion to the next generation (e.g. De Hoon & van Tubergen, 2014; Drouhot, 2021; Guveli et al., 2016a; Molteni & van Tubergen 2022).

As well as shaping the distribution of religious affiliation, migration is also associated with levels of religiosity. Migrants tend to come from less secure societies, which Norris and Inglehart (2011) have shown are associated with higher levels of religiosity. Migrants and their children tend to be more vulnerable and in more marginal positions within Western societies (Heath et al., 2008; Brynin & Guveli 2012); and Immerzeel and van Tubergen (2013) have illustrated the sensitivity of religiosity to insecurity within Europe.

Migrating to an unfamiliar environment with different lifestyles, values and behaviour can create the need for migrants to reformulate and rethink their religion and religious identities to make sense of their new settings (Diehl & Koenig, 2013). Religion is used as a source of support in the process of migration itself (Durand & Massey, 1995; Hagan, 2006; Hagan & Ebaugh, 2003). On the other hand, migration to secular societies can disrupt religious practice and expression of religiosity (Connor, 2008).

Migration encompasses risks, which, on arrival, can engender a need to search for religious networks to assist in daily life and faith (e.g. Cadge & Ecklund, 2007; Levitt, 2007; Palsetia, 2006; Wuthnow & Offutt, 2008). Historically, religious organizations have helped migrants to survive and rebuild their ethno-religious identity in new and challenging contexts (Herberg, 1955; Park & Miller, 1921; Diehl & Koenig 2013; Koenig et al., 2016). Religious institutions and organizations thus play a crucial role in supporting and integrating migrants and their offspring in destination societies. These organizations can also foster the (reformulated) religious commitment of migrants and the second generation in the host society (Voas & Fleischmann, 2012; Yang & Ebaugh, 2001).

Migrant religiosity thus has the potential to reshape the religious landscape in Europe both in terms of religious pluralism and religious expression. Even in an increasingly secular context, increasing migrant religiosity could stimulate revival or maintenance of religious engagement among natives. Given the strong influence of the ‘myth of Christian Europe’ (Adida et al., 2010) on attitudes and conceptions of the nation, increases in religious expression could also represent a reaction to recent perceived challenges to national identity represented by migration (Castles, 2012).

### Religiosity Among the Second Generation

There is mixed evidence on the patterns of religiosity in the second generation (Chen & Park, 2019). Studies of European migrant groups in specific countries have tended to report religious decline across generations (e.g. Maliepaard et al., 2010b; Guveli & Platt, 2011; Beek & Fleischmann, 2020; Drouhot, 2021), though, again, much of this research has focused on Muslims. US research has shown a mixed picture. Alanezi and Sherkat, (2008) found that the second generation had higher rates of participation than migrants; but Chen and Park, (2019) found the opposite, though with distinctive patterns across different affiliations, and with particularly high rates of retention among second-generation American-Asian protestants. Integration theory (Durkheim, 1963 [1952]) and expectations of the norms propagated through education, imply that the second generation will take on their secular society’s values and behaviour. But, compared to natives, religious identification can provide an important source of identity for those born of migrant parents, but brought up in a secular country and with weaker ethno-cultural affiliations (Guveli, 2015; Jacobson, 1997). That is, greater distance from ethnic identities may serve to reinforce transnational religious identities (Drouhot, 2021; Ehrkamp, 2005; Wuthnow & Offutt, 2008), which can provide significant points of connection across generations. Religious identity can also prove especially salient in the face of exclusion or rejection by the majority (Connor, 2010; Drouhot, 2021; Guveli, 2015; Molteni & van Tubergen, 2022; Platt, 2014). However, forms of expression may differ as the second generation reinterpret their religion in the destination country context.

For those who maintain a religious affiliation, these processes may lead to greater expressions of religiosity compared to natives of the same nominal religion (cf. Chen & Park, 2019). However, there may also be variation across affiliations, as migrants face different levels of marginalization and exclusion. For example, Muslims face particularly high rates of discrimination (Adida et al., 2010, 2016; Strabac & Listhaug, 2008), which may promote greater investment in religiosity as a resource (Hirschman, 2004). Conversely, those migrants affiliated to the dominant or historical religion of the country of destination, may more easily assimilate to the lower levels of religiosity of their native counterparts (cf. Connor, 2009). We might therefore anticipate variation across the second generation of different religious affiliations. Research on Muslims has indicated high and rather stable levels of religious affiliation into the second generation (e.g. Manning & Roy, 2010; Voas & Fleischmann, 2012), which extends to dimensions of religiosity (e.g. Simsek et al., 2019). But we have little insight into whether the religiosity of those affiliated to other religions or religious denominations in Europe shows different patterns.

### Over Time Trends

Trends in religiosity of migrant groups over time, rather than across generations (e.g. Jacob & Kalter, 2013) or within specific groups of individuals (e.g. Simsek et al., 2019), are not currently well understood. There are reasons why we might expect both increasing and declining religiosity among those of migrant origin across the period of study. A higher degree of religious organization for various religions now exists in European destination countries since the new arrivals of the 1950s. Migrants invest in creating social, cultural and religious space for themselves and for their group in the host country (Dustmann, 2008; Guveli, 2015; Herberg, 1955; Diehl & Koenig 2013). The need to create institutions is more pressing if the religion of the newly arrived groups differs from that of the host society, because they cannot make use of existing institutions—such as places of worship. When the size of an ethnic or religious group becomes large enough, it will establish its own social, cultural and religious institutions (Breton, 1964; Guveli, 2015). This requires time and expertise, networks and followers; that is, an entire social and cultural infrastructure. Increases in the size of specific migrant groups and better ethno-religious infrastructure then provide greater opportunities to observe and manifest religion.

At the same time, exposure to an increasingly secular environment in most of the European societies will increase the probability that migrants and the second generation will gradually adopt a more secular lifestyle. Continuing new migration flows, however, render first-generation migrants a dynamic group in terms of both their composition and their religious devoutness. As new migrants bring higher levels of religiosity with them or come from more religious contexts, the stock of migrants may become more resilient to processes of secularization.

In addition, even apparently secular Western European states are often imbued with specific religious symbolism, assumptions and privileges, conceptualized as ‘vicarious religion’ by Davie (1994, 2000). States are built upon religious symbols, motifs and understandings, which are translated into–but are nevertheless essential to–secular modern nation-building projects (Adida et al., 2010; Guveli & Platt, 2011). Such processes are themselves dynamic, as the religious roots of a nation may be reasserted in nationalist movements (Spohn, 2009). Newly arriving migrants or the second generation are therefore not simply faced with a blank or secular context but a religious one which, by challenging coherence with their beliefs, may reinforce them. Assimilation in such contexts implies not only the gradual abandonment of a particular belief system, but the tacit or passive acceptance of an alternative one. Conversely, increasing diversity in the religious landscape and high levels of religious observance among newcomers might also revitalize historical religious denominations in destination countries. We therefore aim to shed light on the trends ensuing from these different dynamics.

## Data, Variables and Method

We use the European Social Survey (ESS) as the most suitable data for our research aims. The ESS has been carried out since 2002. It is designed to collect information on the attitudes, beliefs and behaviours of representative samples of the participating countries and enable analysis of stability and change over time.

The ESS has been extensively used for studies of migrants within Europe, including for studies of the religiosity of migrants, and for comparisons of migrants with natives (e.g. van Tubergen & Sindradóttir, 2011; Alekxynska & Chiswick 2013; Immerzeel & van Tubergen, 2013; Guveli, 2015; Guveli & Spierings, 2021; Platt et al., 2022; Molteni & van Tubergen, 2022). For our purposes, it has two unique strengths: (1) the questions are asked the same way in all countries and (2) they are asked the same way in all rounds, making it possible to investigate patterns across Western European countries between 2002 and 2018.^1^ The ESS is unique among social surveys in its potential to investigate trends in the religiosity of migrants and natives, as it has repeated the same questions in all biennial rounds.

We use nine rounds (2002, 2004, 2006, 2008, 2010, 2012, 2014, 2016 and 2018) of the ESS (ESS 2019), and information from 15 Western European countries.^2^ These countries are those which have experienced longstanding or increasing migration and are most extensively analysed in the European migration literature (Geddes & Scholten, 2016) and in studies of Muslims in Europe (Statham & Tillie, 2018). In some years, the question on religious affiliation was not asked in certain countries. We therefore exclude those specific country-rounds; but we include information from the countries for other years. The countries in our sample participated at least five times across the period, and the average participation rate was nearly eight times.^3^

Due to small numbers of Jews of any generation across our sample, we exclude those cases. This leaves us with a pooled sample of 238,350 cases for which we have complete information across all dependent variables and covariates on 226,301 cases, with 5 per cent of respondents having missing values for any of our measures.

### Dependent Variables

Our dependent variables are: prayer, attendance and subjective religiosity. Glock (1962) highlighted that religiosity could be evaluated according to distinctive dimensions. These three measures have been collected in a range of studies and are typically analysed separately (e.g. De Hoon & van Tubergen, 2014; van Tubergen & Sindradóttir, 2011). This is because they are conceptually distinct, and they may differ across religions and between migrants and natives in their salience (Guveli, 2015).

Prayer is measured with the question ‘Apart from when you are at religious services, how often, if at all, do you pray?’, with response categories ranging from every day (coded 6), through more than once a week (5), once a week (4), at least once a month (3), only on special holy days (2), less often (1) to never (0). Frequency of attendance at places of worship is captured with the question, ‘Apart from special occasions such as weddings and funerals, about how often do you attend religious services nowadays?’ and utilizes the same seven response categories. Given sparse responses in the most frequent categories, we recode to a five-category variable with the most frequent category of weekly or more often. Subjective religiosity is asked as: ‘Regardless of whether you belong to a particular religion, how religious would you say you are?’, with responses on a scale from 0 (not at all religious) to 10 (very religious). Importantly, all three questions are asked regardless of whether or not the respondent states a particular religious affiliation. We can thus capture the religiosity of the non-affiliated (Davie et al., 2017) and compare the religiosity of the affiliated with the nominally non-affiliated, providing us with a consistent baseline category across all contexts.

### Independent Variables

Religious affiliation is measured with the question ‘Do you consider yourself as belonging to any particular religion or denomination?’. We allocate those who answer ‘no’ to the category of ‘no religion’. For those who answer yes, the options provided are: Roman Catholic; Protestant; Eastern Orthodox; Other Christian denomination; Jewish; Islamic; Eastern religions; Other non-Christian religions. As noted, we exclude the small numbers of Jewish respondents from our sample. We combine Eastern religions and Other non-Christian religions into a single ‘Other category’. Due to the small size and heterogeneity of this category, while we include it in analyses for completeness, we do not discuss it further.

To identify time trends, we include ESS round (year) from 1 (2002) to 9 (2018) in all models. Inspection of patterns of religiosity over time across the whole sample indicate that time trends are approximately linear (Supplementary materials, Table S1). However, we tested whether this was the case for the different generations and religious affiliations, by employing ESS round as a nominal variable interacted with religious affiliation and generation in our main models. The time trends were consistent with those in the linear specification, though the figures derived from these alternative analyses demonstrated that there was somewhat greater ‘noise’ for those of Orthodox affiliation. We provide the graphs derived from models with this alternative specification in the Supplementary Materials, Figure S1, which demonstrates the comparability with our linear specification for the time trend. Given the consistency between the models, in the interests of parsimony we include ESS round as an interval variable in our main models. We conducted additional analysis to test the sensitivity of our results to the inclusion/ exclusion of any given wave. Results were robust to these alternative specifications.

For migrant status, we distinguish natives, migrants (first generation), and children of migrants (second generation). The ESS provides information about the country of birth of the respondents, and their father and mother, which allows us to compare Europeans living in their native land with first-generation migrants and the second generation of migrant origin. We define natives as those where neither the respondent nor either parent was born abroad; migrants (first generation), where the respondents and their parents were born abroad, and second generation, where at least one of the respondent’s parents was born abroad but the respondent was born in the survey country.

In the first, 2002, round of the ESS, only the continent of the father’s and mother’s birthplace was asked. However, we used the first and second language spoken at home together with the father’s and mother’s continent of birth to identify the country of their birth. Cross-checking this method on other ESS rounds, the correlation between parents’ country of birth and continent of birth and first and second language spoken at home was 0.93. The share of natives in our sample overall is around 84 per cent, while the share of migrants is around nine per cent and the share of the second generation is around seven per cent, though with some variation across countries. Table S2 in the Supplementary materials provides information on the shares of the different migrant generations by country.

### Control Variables

Given that migrants and the second generation may be distinctive on some characteristics, we include them in our analysis so that we can identify patterns of religiosity net of these compositional factors. First, women are typically more religious than men (De Vaus & McAllister, 1987), we therefore include a variable coded men (0) and women (1). Marriage patterns also vary between those of migrant origin and native populations, and marriage tends to be associated with higher levels (e.g. van Tubergen, 2006) or greater retention (Uecker et al., 2007) of religiosity. Married people are typically more religious than single or separated/divorced (van Tubergen, 2006). We therefore code individuals’ marital status as married/ cohabiting/in legal partnership (0), divorced/widowed/separated (1), or never married (2).

Age tends to be positively associated with religiosity. Whether this represents a consequence of ageing or is a cohort effect has been debated (Crockett & Voas, 2006). We cannot distinguish between these competing positions, but either way, it is important to capture age, especially as the second generation tends to be younger on average than the migrant generation or natives. We include age in years.

While some studies point to a negative relationship between education and religiosity (e.g. Guveli & Platt, 2011), some others do not find this (Albrecht & Heaton, 1984: Campbell & Curtis, 1994; Te Grotenhuis & Scheepers, 2001). The direction of this relationship might be different for distinct migrant and religious groups. Moreover, migrants also tend to be more highly selected on education than their non-migrant counterparts (e.g. Feliciano & Lanuza, 2017; Bayrakdar & Guveli, 2021; Guveli & Spierings 2022). We thus include education measured in years, since this is the only feasible way of proxying educational attainment across a diverse range of countries.

Whether or not the respondent is in paid work is likely to be an important influence on their degree of religiosity (Immerzeel & van Tubergen, 2013). We therefore include a measure of whether (1) or not (0) the respondent was in paid work.

Descriptives of all variables broken down by migrant status are provided in Table 1.

**Table 1:** Descriptive statistics of sample

	Natives				First generation				Second generation				All			
	Mean /%	sd	min	max	Mean /%	sd	min	max	Mean /%	sd	min	max	Mean /%	sd	min	max
Prayer	2.2	2.4	0.0	6.0	3.0	2.5	0.0	6.0	2.1	2.4	0.0	6.0	2.2	2.4	0.0	6.0
Attendance	1.4	1.4	0.0	4.0	1.6	1.5	0.0	4.0	1.2	1.3	0.0	4.0	1.4	1.4	0.0	4.0
Subjective religiosity	4.4	2.9	0.0	10.0	5.4	3.1	0.0	10.0	4.5	3.1	0.0	10.0	4.5	3.0	0.0	10.0
Age	49.6	18.5	18.0	100.0	44.1	16.1	18.0	100.0	42.4	18.1	18.0	100.0	48.6	18.5	18.0	100.0
Women	52.0				53.1				52.3				52.1			
Survey round	5.0	2.6	1.0	9.0	5.5	2.5	1.0	9.0	5.3	2.6	1.0	9.0	5.1	2.6	1.0	9.0
Years of education	12.5	4.3	0.0	30.0	13.1	4.5	0.0	30.0	13.1	3.8	0.0	30.0	12.6	4.3	0.0	30.0
In paid work	53.0				57.3				55.7				53.6			
*Marital status*																
Married	51.5				55.5				40.3				51.1			
Divorced/widowed	18.5				17.2				16.3				18.3			
Never married	30.0				27.4				43.3				30.6			
*Religious affiliation*																
No religion	42.1				36.1				47.5				41.9			
Catholic	35.1				26.3				26.8				33.7			
Protestant	21.2				10.1				13.5				19.7			
Orthodox	–				5.1				1.0				0.5			
Other Christian	1.1				3.2				1.6				1.3			
Islam	–				15.5				8.2				2.0			
Total (N)	190,634				20,577				15,090				226,301			

*Source* ESS Rounds 1 (2002)–9 (2018)

### Analytical Approach

While our primary interest is in identifying how our dimensions of religiosity are patterned across religions and over time for those with different religious affiliations, increasing secularization could also arise through changing rates of those affiliating to no religion as well as through their religious expressions. We therefore first plot the extent to which there is variation in those holding no religious affiliation over time. We then move on to investigate the patterns across our three dependent variables to address our research questions.

In line with existing approaches, we treat our three dimensions of religiosity as continuous (Aleksynska & Chiswick, 2013) and estimate a series of nested ordinary least square (OLS) regressions for each of the dependent variables to isolate the association of migrant status and religious affiliation with religiosity, taking account of compositional differences. In final models we interact, first, migrant status, and then, combined religious affiliation and generation with the ESS round, to identify time trends. For ease of interpretation, we present results from these final models graphically, while providing the full sets of regression coefficients in the Supplementary materials (Tables S4–S6). In commenting on our results, we only refer to differences that are statistically significant at the 5 per cent level.

There has been a lively debate on appropriate models to apply using cross-national surveys such as the ESS (Bryan & Jenkins, 2016; Te Grotenhuis et al., 2015). Given the small number of units with which we are concerned, using multilevel models is likely to be inappropriate. Moreover, we are not concerned with identifying the contribution of contextual factors but with describing patterns of religiosity for those of different religions across Europe (or more specifically Western European countries). That is, rather than attempting to explain how far religiosity can be attributed to (differential) composition of migrants according to country of origin, we are interested in ascertaining if such (changes in) composition impacts the destination context in relation to patterns of religiosity. To account for all those country-level factors that might be associated with both the distribution of migrants and religious groups across countries and the levels of religiosity, we incorporate destination country fixed effects in all our models (Clarke et al., 2015). This absorbs all those sources of variation at the country level that might mediate the relationship between our key independent and dependent variables. Such use of country fixed effects is a conservative approach (Bryan & Jenkins, 2016) that leaves us able to identify the aggregate patterns we are interested in.

We conducted additional analyses to test the robustness of our results to different specifications. First, we repeat the analysis only on those countries within our sample that both participated in all ESS rounds and provided information on religious affiliation at every round (Belgium, Switzerland, Germany, Spain, Ireland, Netherlands, Norway, Portugal, Sweden). Second, to identify if the specific religious context of the destination country is relevant to our results, we restrict our analysis to those countries that have either Protestantism or Catholicism as a dominant religion (Germany and the Netherlands have both strongly Catholic and strongly Protestant regions so can be considered in both groupings). Third, to take account of any undue influence of any one country context on our result, we repeat our analysis excluding each country in turn from our sample. This exclusion approach also addresses the undue influence of countries which have higher non-response, or where there appears to be potentially a less comprehensive coverage of migrants (cf. Platt et al., 2022). Table S3 in the Supplementary material summarizes estimates of migrant populations for our countries from OECD data compared with our sample.

One concern with the use of ESS for the study of migrant populations is the potential differential selection of migrants into the sample. One potential source of bias in the ESS is that it does not translate into minority languages unless they are spoken by five per cent or more of the population. If religiosity is correlated with inclusion, for example if more religious respondents are less likely to speak the host society language, this could bias our results for the first-generation immigrants. As a robustness check, we therefore re-estimate our models excluding all those who arrived in the last five years, on the basis of the well-attested findings on language assimilation over time (e.g. Carliner, 2000; Akresh, 2006; Aleksynska & Algan, 2010a). Isphording (2015) shows migrants continue to learn the destination country language even after 20 years, but the first five years are an important milestone for them to acquire a good level of language fluency. This exclusion avoids the issue of potential differential selection on language (and therefore potentially on religiosity) across countries. The main conclusions do not change when conducting these different analyses (available on request). The more limited coverage when restricting to Catholic and Protestant countries does, however, provide less leverage for comparisons across affiliations.

## Results

### Levels of Religious Affiliation

**Fig. 1:**
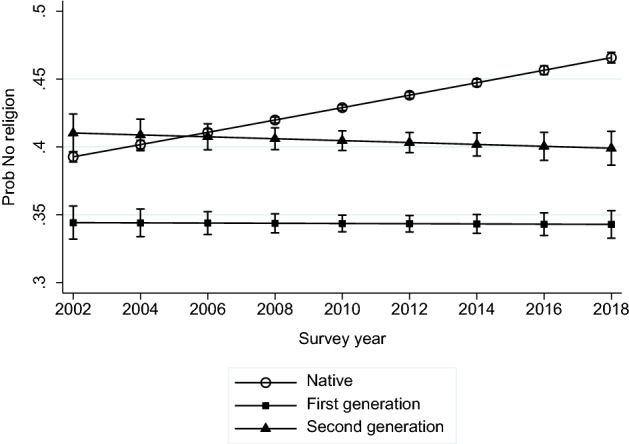
Probability of not affiliating to any religion over time, by migrant status, *Source*: ESS marginal probabilities from logistic regression model controlling for age, sex, marital status, educational level, and country fixed effects

Before turning to our main analysis, we first address the question of how far secularization is taking place not in forms of religiosity but in terms of adherence to any religion. Unadjusted levels of non-affiliation are around 42 per cent for natives, 36 per cent for migrants and 48 per cent for the second generation, in line with its more youthful profile (see Table 1). Put differently, across all groups over half of all respondents affiliate to a religion. Regressing the probability of non-affiliation on compositional factors and migrant generation and controlling for country fixed effects shows that there was a statistically significant increase in secularization over time, but that this was driven by the native population. Figure 1 illustrates that, for adjusted estimates, the probability of expressing no religious affiliation was significantly lower for migrants compared to natives, while for the second generation the rates were much more comparable to natives but diverged over time. This indicates that, for natives, migrant religiosity is not associated with a reactive increase in affiliation.

### Patterns of Religiosity

**Fig. 2:**
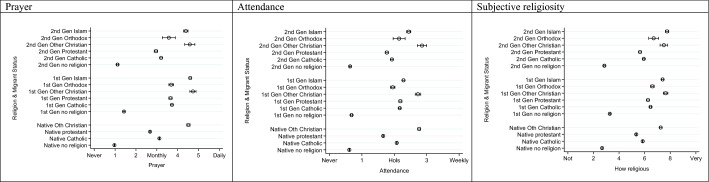
Differences in religiosity by religious affiliation and migrant status, with 95% confidence intervals, *Source*: ESS Rounds 1 (2002)–9 (20,018); estimates control for age, sex, marital status, educational level, ESS round and country fixed effects

We now turn to address the primary question of how far natives, and those of migrant origin differ in their religiosity. Adjusting for the full set of individual controls, country fixed effects and survey year, Fig. 2 shows how religiosity differs by religious affiliation and generation (see also Model 3 in Tables S4–S6 in the Supplementary materials). Looking at prayer, in line with our expectations migrants tend to be more religious than natives and, interestingly, this also applies to migrants with no religious affiliation, even though rates of religiosity are substantially lower among those without an affiliation. The exception to this general pattern of greater religiosity among migrants is the ‘Other Christian’ denominations, who have ubiquitously high levels of religiosity. These more evangelical denominations may themselves reflect particularly engaged or committed Christians compared to the more ‘cultural’ Christianity associated with historically dominant religions (Davie, 1994). Partly in line with our expectations, second-generation Protestants and Catholics show convergence to their native counterparts. By contrast, Muslims, Other Christians and Orthodox have levels of religiosity that are comparable to their first-generation counterparts.

The findings for measures of attendance and subjective religiosity are largely similar, though second-generation Muslims have higher levels of subjective religiosity than their first-generation counterparts, while the levels for Orthodox and Other Christians are comparable across the generations. It is only on this measure that Muslims have higher levels of religiosity than Other Christians. This may be indicative of a movement towards a more general Muslim identity that is not so strongly embedded in specific behaviours (Jacobson, 1997; Platt, 2014).

In summary, with a small margin, Other Christians and Muslims show the highest levels of religiosity for all three measures of prayer, attendance and subjective religiosity. The relatively high levels of religiosity among Muslims are consistent with other research (e.g. Drouhot, 2021; Guveli, 2015; Simsek et al., 2019; Voas & Fleischmann, 2012), but we see here that Muslim religiosity is comparable to that of the Other Christian category, and not far removed from Orthodox Christian patterns. As Voas and Fleischmann (2012) note, Muslim exceptionalism may be overstated. Given that differences by migrant status tend to be small, the major cleavage is between those with no religious affiliation and those with any.

In relation to our first question, therefore, our findings support expectations that religiosity is higher among migrants. We find limited convergence in the second generation, though more so for those sharing dominant European religions of Catholicism and Protestantism. In terms of the different measures, we find no compelling evidence that second-generation practices differ from the first generation.

### Evolving Trends: Religiosity over Time Between 2002 and 2018

**Fig. 3:**
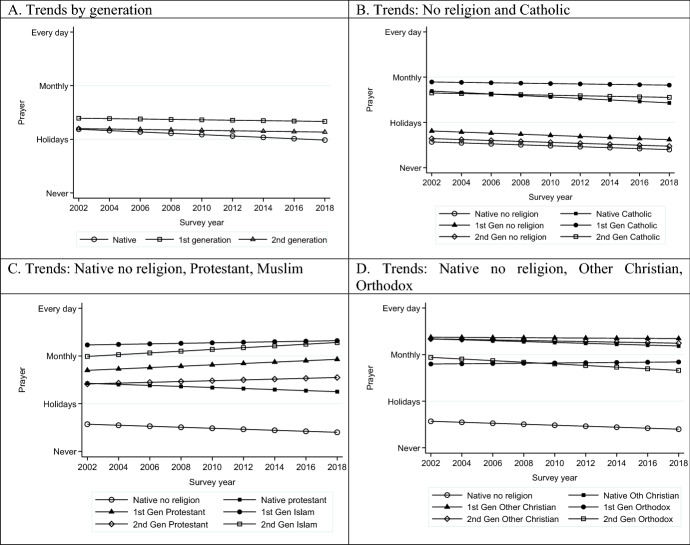
Trends in religiosity by migrant status and by religious affiliation and migrant status: Prayer, *Source*: ESS Rounds 1 (2002)–9 (2018); estimates control for age, sex, marital status, educational level, and country fixed effects. Panel A controls additionally for religious affiliation

**Fig. 4:**
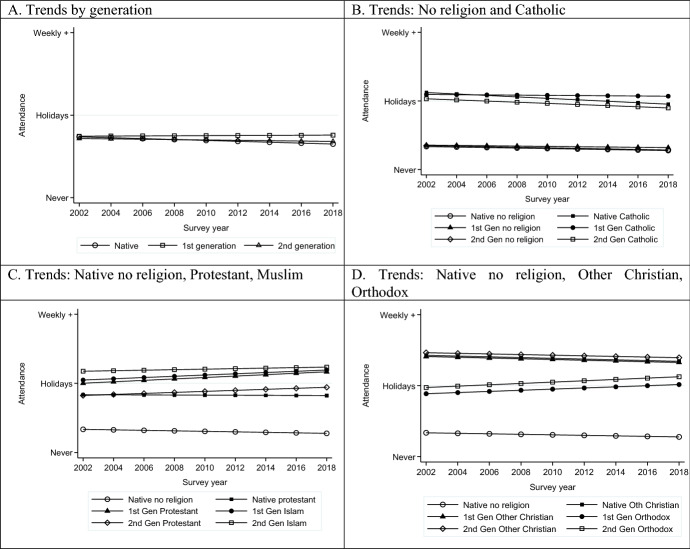
Trends in religiosity by migrant status and by religious affiliation and migrant status: Attendance, *Source*: ESS Rounds 1(2002)–9 (2018); estimates control for age, sex, marital status, educational level, and country fixed effects. Panel A controls additionally for religious affiliation

**Fig. 5:**
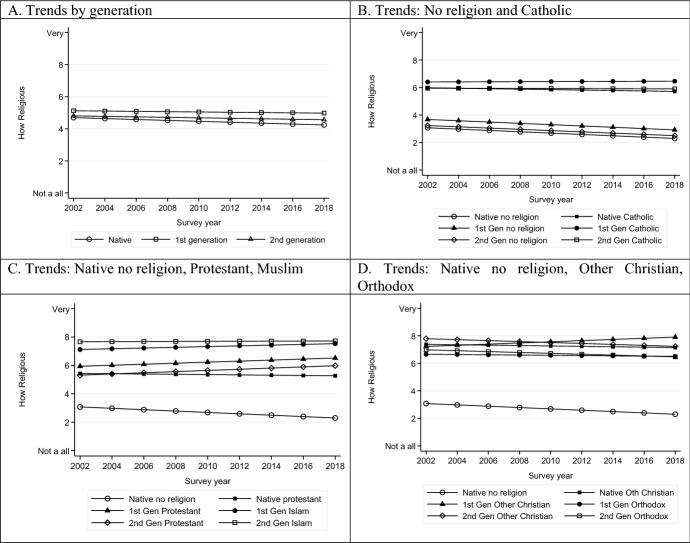
Trends in religiosity by migrant status and by religious affiliation and migrant status: Subjective religiosity, *Source*: ESS Rounds 1(2002)–9 (2018); estimates control for age, sex, marital status, educational level, and country fixed effects. Panel A controls additionally for religious affiliation

Over the 2002–2018 period, there was a small but significant decline in religiosity across all three measures: praying, attendance and subjective religiosity (see Supplementary Materials Tables S4–S6). Combined with the small increasing trend in those affiliating to no religion, this supports the expectation of increasing secularization of Western European societies over time, rather than reaction to migration. In Figs. 3, 4, 5, we explore further how this trend plays out for different religious affiliations and generations. Each figure shows the time trends over the period for natives, first generation and second generation, followed by three figures to show the trends for each religious affiliation and generation, referenced against natives with no affiliation. All trends are based on full models controlling for compositional factors and country fixed effects. Panel A of all three figures illustrates how limited the differences are by migrant status in aggregate. This reflects the substantial shares of non-affiliated, who have low levels of religiosity, among natives and migrants alike.

Turning to Panels B-D, the no religion reference group has a downward trend in terms of religiosity for all three measures, showing declining religious behaviours and commitment, even among those who do not profess a religion. This is counter to claims of non-affiliated spirituality replacing formal religion. When combined with the information on increases in non-affiliation among the native population, it again lends no support to religious expression being stimulated as a response to migration.

Among those affiliated to religions there are, however, some differences in trends. Native, first- and second-generation Catholics in Western Europe show declining prayer and attendance, paralleling the patterns for non-affiliated (Figs. 3B, 4B). This might suggest a process of adaption to local levels of religiosity for historically established religions in line with Herberg’s (1955) theory (see also the discussion in Chen & Park, 2019). However, when it comes to subjective religiosity, they diverge from this secularizing trend. This may be understood as believing rather than belonging in line with Davie’s (1994) argument, and subsequent research on the greater salience of personal belief (Davie et al., 2017).

By contrast, native, first- and second-generation Protestants show a stable or rising trend in all measures of religiosity over time (Figs. 3C, 4C and 5C), leading to divergence from the non-affiliated. The rising trends for first- and second-generation Protestants is comparable to that for first- and second-generation Muslims across all three measures even if at slightly different levels. Among Orthodox Christians (Figs. 3D, 4D, 5D), by contrast, increasing levels of religiosity over the period are observed for the first generation but not for the second generation. The latter, despite substantially higher levels, experiences a parallel decline to the non-affiliated. This may suggest that the first-generation patterns are attributable in part to changing composition, while the second generation may be on a slow journey of convergence to native levels of religiosity. Finally, Other Christians are marked by the highest levels of religiosity, but, nevertheless, in the second generation are seeing relative increases. We therefore observe a mixed picture of stability, decline and revival in the frequency of prayer and attendance and intensity of subjective religiosity.

What is notable about these findings is that, other than the experience of Catholics who show declines in attendance and prayer but not subjective religiosity, the patterns are remarkably consistent across the different measures. This suggests that there is not some form of exchange across different domains of religious expression but that they have the same underlying correlates. The second notable point is that the second generation of not only Muslims but also Protestants and Other Christians are experiencing increases in religiosity over the period. This is the first clear evidence we have come across for religious revival in recent decades among minority religions.

The situation of second-generation Protestants would seem to be distinctive. Their significantly greater levels of religiosity over time compared to their native counterparts is not in line with the downward assimilation to native levels and greater secularization that might be expected for such mainstream affiliates.

While not only the gaps between religions but also the changes over time are relatively small, this is hardly surprising over a short period of sixteen years. Any more dramatic change would be rather implausible. Instead, our findings indicate the directions that religious landscape of Europe may be heading in, especially as the children of migrants make up increasing shares of the population. For Muslims and those of Other Christian denominations, religiosity looks likely to remain high. At the same time, for Protestants, who have seen declining rates of religiosity in their historical bases (Smith & Kim, 2005), the children of migrants may provide some reversal. The generally secularizing trends of the non-affiliated will temper the amount of aggregate change that can be expected, even as there is increasing divergence between those affiliated and not.

## Discussion and Conclusions

Making the most of the distinctive features of the ESS, we have explored patterns of religiosity across migrant generations compared to native populations for three different forms of religiosity. We aimed to enhance understanding of how migrant and second-generation religiosity differs from or is similar to native-born with no religious affiliation. We investigated not only whether levels of religiosity differed by migrant status, after adjusting for relevant individual characteristics, but also whether there were any distinctive trends in religiosity over a period of sixteen years that has been marked by increasing political and public attention to migrant religion and to the place of religion in national belonging.

We found differences in religiosity across migrant generations, with migrants tending to be more religious than otherwise comparable natives, and with the second generation in the middle. But when we looked within religious affiliations, we found these differences were rather small, and in some cases non-existent or reversed. Given the ways in which native and migrant populations are likely to differ in terms of the security and resources offered by religion, it was also striking how slight the distinctions between religious denominations were. Muslims and Other Christians showed greater religiosity than those affiliated to traditional Western European religions (Catholics & Protestants). But as our figures clearly illustrated, the largest gap is between those with no affiliation and the rest. While it is unsurprising that the ‘nones’ (Lim et al., 2010) have lower patterns of religiosity, they have still been argued to maintain some involvement with the symbolic and expressive aspects of religion. Even among this group of non-affiliates, prayer and subjective religiosity tends to be higher among migrants and the second generation, suggesting migrants remain more attached to the symbolic aspects of religion even if they lack—or have lost—a specific faith.

Our other key finding relates to change over time. We currently have little insight into the religiosity of the second generation as it is evolving relative to native populations in countries of destination, though the second generation will contribute to shaping the future religious landscape of Europe. We speculated that, as an alternative to convergence, we might find increasing religiosity among the second generation, as they seek reinforcement and support not only in an often secular but also in a more polarized context. Our results supported this contention for Other Christian and Muslim trends. Religion has been conceived of as a specific resource for migrants, particularly in the early days of settlement (Hagan & Ebaugh, 2003; Phillips et al., 2007) when institutional forms of religion can aid adjustment. Our findings support this for the second generation, who may experience ‘blocked acculturation’ (Adida et al., 2016; Wimmer & Soehl, 2014) or a discriminatory environment (Di Stasio et al., 2021).

Prior to our study, there has been little general evidence for ‘religious revival’. However, despite the attention paid to Muslim religiosity within the literature, we note that the evidence for religious revival is matched among migrant and second-generation Protestants, whose religiosity over the period increased significantly more than that of their native counterparts across all three measures, potentially reversing trends towards greater secularization in this group. While, as noted, the increases were small in magnitude, they indicate an under-researched aspect of migrant religiosity that deserves greater scrutiny.

Our initial evidence for religious maintenance and revival in the second generation merits further consideration of its drivers and consequences. Brought up in a typically secular context and with, in most cases, greater opportunities and resources than their first-generation forbears, traditional expectations would be for gradual secularization. However, rich institutional religious resources developed by the migrant generation may provide welcome sources of meaning and support for the second generation (Breton, 1964; Guveli, 2015; Herberg, 1955), as they face what may feel like an increasingly disorientating and potentially hostile world. Religion can function as a protective environment against those facing discrimination and marginalization (Chen & Park, 2019; Lancee, 2021; Connor, 2010; Adida, 2016; Heath & Di Stasio, 2019; Van Ballegooij & Moxom, 2018b), which can be more keenly felt by second-generation Muslims (Drouhot, 2021; Guveli, 2015; Platt, 2014). This explanation is, however, unlikely to apply to the increasing trend of religiosity of the second-generation Protestants, which needs further scrutiny in future research. How religious attachment is fostered or sustained, including through kin and peer networks is a fruitful area of study for the future. We suggested that natives affiliated to majority religions in Europe might react to migrant religiosity and become increasingly interested in their ‘Christian culture’ (Adida et al., 2010). While Catholic attendance and prayer declined over time, subjective religiosity did increase. These different trends across the two main Christian denominations in Western Europe are intriguing and suggest that we may need to refine understandings of Christianity within Europe.

The composition of first-generation migrants is clearly dynamic, and the origins and reasons for migration are changing over time, potentially impacting the religiosity of the migrant population in Europe. While a limitation to our study is that we are unable to plot intra-individual processes of change, conversely, the ability to capture the changing profile of European migration was a key part of our interest in plotting religiosity of the migrant generation. The repeated cross-sectional ESS data is well suited to this. For the second generation, such compositional change is less relevant. It cannot help to account for the observed increasing religiosity among some of the second generation. If that can be attributed to inter-individual change, as seems likely, analysis of its potential drivers would benefit from cross-national longitudinal data.

A limitation of our study is that the first-generation migrants are likely to be a selected group and may not be representative of the population in the countries we study. This is because the ESS includes respondents who are able to answer the survey questions in the destination country language. This might result in greater inclusion of more integrated first-generation migrants who might be less religious than those who are not included in the survey. This would bias our estimates of migrant religiosity downwards. While we incorporated a range of checks to address this issue, we cannot eliminate the possibility of differential inclusion by level of religiosity.

The answer to our question as to whether migration is affecting the religious landscape of Europe is, then, twofold. There remain high levels of religious affiliation in Western Europe. Over half of Western Europeans still affiliate with a religion, and migrants do not enter a context that is devoid of religious customs and practices. Nevertheless, among those who do not affiliate to a religion, attachment to the cultural practices and commitment associated with formal religion appears to be loosening its hold. As a result, we find that religiosity has been declining over the course of the period between 2002 and 2018 for all dimensions of piety; and the historical trend of secularization continues despite changes to national composition (Spohn, 2009; Pew Research Centre 2018a). Since this derives more from lack of conviction among non-affiliated than by loss of faith among those with religious affiliations, there are also moves towards divergence in religiosity both between those with no and those with any affiliation, and between natives and people of migration background.

### Supplementary Information

Below is the link to the electronic supplementary material.

Supplementary file1 (PDF 752 KB)

## Footnotes

1 All migrant-related studies have limitations. Specialist migrant surveys are mostly sampled from high migrant concentration areas by either screening the regions or on the basis of address registers. Many explicitly sample from cities only. These surveys therefore tend to exclude migrants or those of migrant origins who live outside high-concentration and segregation areas and might therefore be more integrated (and perhaps more secular) than those in segregated and concentrated areas. Response rates are a concern across studies, given that while specialist studies typically face low response rates, substantially lower than the overall rates in the ESS (Font & Mendez 2013), immigrants and the second generation may face lower response rates than the average in the ESS given that response rates are correlated with socio-economic status, and migrant groups tend to be concentrated among those with lower socio-economic status. There are, moreover, only a few migrant surveys (TIES (Crul et al., 2012), 2000 Families (Guveli et al., 2016a, 2016b), SCIP (Diehl et al., 2016)) that employ a cross-national research design enabling comparative research on religiosity across European countries. These face their own sampling challenges, as well as limiting the potential for comparison to a small number of countries. Other comparative studies, such as EURISLAM (Hoksbergen & Tillie 2012), focus specifically on Muslims, or on particular demographics (e.g. CILS4EU (Kalter et al., 2016) only covers schoolchildren). Some comparative migrant surveys, such as the SCIP survey also do not include native comparators.

2 The countries are: Austria, Belgium, Denmark, Finland, France, Germany, Ireland, Italy, Netherlands, Norway, Portugal, Spain, Sweden, Switzerland, UK.

3 We carry out supplementary analysis on the nine countries that participated in all nine rounds. The conclusions remain the same (available on request).
