# Supplementary material for: Religiosity of Migrants and Natives in Western Europe 2002–2018: Convergence and Divergence
Source: Eur J Popul. 2023 Mar 23;39(1):9. doi: 10.1007/s10680-023-09660-9 (PMC10036697; doi:10.1007/s10680-023-09660-9)
Supplement: Supplementary file 1 — Supplementary file1 (PDF 752 KB) [file 10680_2023_9660_MOESM1_ESM.pdf]

Supplementary Tables and Figures

Table S1: Linearity of time trends for each dependent variable

ESS Round (Ref =1)	Prayer	Attendance	Subjective religiosity
2	-0.029	0.024*	-0.072**
	(0.0198)	(0.0118)	(0.0254)
3	-0.113***	-0.025*	-0.151***
	(0.0195)	(0.0116)	(0.0250)
4	-0.151***	-0.063***	-0.222***
	(0.0193)	(0.0115)	(0.0247)
5	-0.234***	-0.108***	-0.339***
	(0.0200)	(0.0119)	(0.0256)
6	-0.261***	-0.120***	-0.294***
	(0.0191)	(0.0114)	(0.0245)
7	-0.279***	-0.112***	-0.419***
	(0.0192)	(0.0115)	(0.0246)
8	-0.352***	-0.158***	-0.423***
	(0.0193)	(0.0115)	(0.0247)
9	-0.393***	-0.193***	-0.507***
	(0.0191)	(0.0114)	(0.0245)
R-squared	0.202	0.164	0.128
N		226,301	

Source: ESS Rounds 1-9, estimates from model controlling additionally for age, sex, marital status, years of education, work status and country fixed effects.

Table S2: Migrant status by country

	Native	First	Second	N (100%)
Austria	82.1	8.7	9.2	12,133
Belgium	79.7	10.5	9.9	15,647
Switzerland	64.8	21.4	13.7	14,828
Germany	83.1	8.7	8.3	24,817
Denmark	89.3	5.4	5.3	11,998
Spain	89.8	8.3	2.0	16,086
Finland	95.4	2.9	1.7	13,754
France	79.3	9.0	11.7	13,329
UK	81.6	10.6	7.8	15,024
Ireland	85.2	10.8	4.1	19,434
Italy	92.5	5.4	2.1	8,257
Netherlands	85.5	7.7	6.8	16,516
Norway	87.1	8.3	4.6	14,394
Portugal	93.2	4.5	2.3	14,477
Sweden	80.4	11.1	8.6	15,607
All countries	84.2	9.1	6.7	226,301

Source: ESS Rounds 1-9

Table S3: Share migrants by comparison with OECD estimates adjusted for working age

	ESS 2004/6 Working age	ESS 20016/18 Working age	OECD 2006 estimates with working age adjustment	OECD 2017 estimates with working age adjustment	Difference OECD-ESS 2006	Difference OECD-ESS 2017
Austria	6.5	11.5	11.7	15.2	5.3	3.6
Belgium	7.9	15.6	9.9	13.3	1.9	-2.3
Switzerland	18.7	28.7	19.2	23.4	0.5	-5.3
Germany	8.1	12.0	10.3	12.4	2.2	0.4
Denmark	5.2	6.8	5.3	8.9	0.1	2.1
Spain	7.6	12.5	9.3	10.4	1.7	-2.1
Finland	2.6	4.4	2.9	5.2	0.3	0.8
France	9.0	10.5	9.1	9.8	0.1	-0.6
UK		15.2	7.5	11.3		-3.9
Ireland	6.7	18.2	11.0	13.4	4.3	-4.7
Italy	1.6	9.8	7.8	8.2	6.3	-1.6
Netherlands	9.3	9.6	8.4	10.0	-0.8	0.5
Norway	7.3	11.5	6.9	11.8	-0.5	0.3
Portugal	4.8	9.0	5.7	6.9	0.9	-2.1
Sweden	10.4	13.5	10.3	14.4	-0.1	0.9

Source: ESS sample estimates (<65) for Rounds 2/4 and 8/9. OECD estimates from Figure 2.1, of OECD (2018) Indicators of Immigrant Integration 2018: Settling In. Paris: OECD, adjusted for estimated 80% share working age (same source).

Table S4: Full estimates from OLS models of frequency of prayer

	Model 1	Model 2	Model 3	Model 4	Model 5	Model 6
ESS round	-0.058***	-0.042***	-0.045***	-0.068***	-0.050***	-0.042***
	(0.0018)	(0.0016)	(0.0016)	(0.0019)	(0.0017)	(0.0027)
1st generation	0.996***			0.673***	0.376***	
	(0.0161)			(0.0378)	(0.0340)	
2nd generation	0.335***			-0.004	-0.009	
	(0.0185)			(0.0415)	(0.0370)	
1st gen#ESS round				0.059***	0.036***	
				(0.0062)	(0.0056)	
2nd gen#ESS round				0.065***	0.034***	
				(0.0071)	(0.0063)	
Native Catholic			2.157***			2.271***
			(0.0121)			(0.0230)
Native protestant			1.710***			1.716***
			(0.0127)			(0.0257)
Native Oth Christian			3.549***			3.529***
			(0.0421)			(0.0869)
Native other religions			2.368***			2.374***
			(0.0670)			(0.1437)
1st Gen no religion			0.459***			0.491***
			(0.0234)			(0.0545)
1st Gen Catholic			2.760***			2.624***
			(0.0273)			(0.0640)
1st Gen Protestant			2.689***			2.160***
			(0.0428)			(0.0988)
1st Gen Other Christian			3.767***			3.571***
			(0.0752)			(0.1823)
1st Gen Orthodox			2.726***			2.405***
			(0.0598)			(0.1561)
1st Gen Islam			3.636***			3.261***
			(0.0350)			(0.0876)
1st Gen Other			3.182***			2.945***
			(0.0699)			(0.1761)
2nd Gen no religion			0.151***			0.149**
			(0.0238)			(0.0542)
2nd Gen Catholic			2.239***			2.144***
			(0.0313)			(0.0686)
2nd Gen Protestant			1.994***			1.613***
			(0.0433)			(0.0940)
2nd Gen Other Christian			3.616***			3.516***
			(0.1242)			(0.2674)
2nd Gen Orthodox			2.610***			2.770***
			(0.1601)			(0.3939)
2nd Gen Islam			3.424***			2.728***
			(0.0554)			(0.1521)
2nd Gen Other			2.762***			2.286***
			(0.1241)			(0.2963)
Nat Cath#ESS round						-0.023***
						(0.0039)
Nat Prot##ESS round						-0.001
						(0.0045)
Nat Oth Christian##ESS round						0.004

						(0.0157)
Nat Oth##ESS round						-0.001
						(0.0251)
1 Gen No rel#ESS round						-0.006
						(0.0090)
1 Gen Cath#ESS round						0.025*
						(0.0107)
1 Gen Protl#ESS round						0.100***
						(0.0169)
1 Gen Oth Chr#ESS round						0.035
						(0.0299)
1 Gen Orthodox#ESS round						0.053*
						(0.0242)
1 Gen Islam#ESS round						0.065***
						(0.0140)
1 Gen Other#ESS round						0.041
						(0.0283)
2 Gen No rel#ESS round						0.000
						(0.0092)
2 Gen Cath#ESS round						0.018
						(0.0117)
2 Gen Protl#ESS round						0.076***
						(0.0167)
2 Gen Oth Chr#ESS round						0.019
						(0.0456)
2 Gen Orthodox#ESS round						-0.028
						(0.0609)
2 Gen Islam#ESS round						0.116***
						(0.0236)
2 Gen Other#ESS round						0.084+
						(0.0475)
Catholic		2.163***			2.163***	
		(0.0112)			(0.0112)	
Protestant		1.727***			1.746***	
		(0.0120)			(0.0120)	
Orthodox		2.650***			2.185***	
		(0.0562)			(0.0574)	
Oth Christian		3.556***			3.474***	
		(0.0355)			(0.0355)	
Islam		3.516***			3.116***	
		(0.0300)			(0.0318)	
Other		2.701***			2.504***	
		-0.0455			-0.0456	
Age	0.025***	0.019***	0.020***	0.025***	0.020***	0.020***
	(0.0003)	(0.0003)	(0.0003)	(0.0003)	(0.0003)	(0.0003)
Women	0.861***	0.722***	0.718***	0.860***	0.718***	0.718***
	(0.0092)	(0.0083)	(0.0082)	(0.0092)	(0.0082)	(0.0082)
Divorced widowed	-0.107***	0.072***	0.068***	-0.106***	0.069***	0.069***
	(0.0127)	(0.0114)	(0.0114)	(0.0127)	(0.0114)	(0.0114)
Never married	-0.272***	-0.082***	-0.061***	-0.271***	-0.060***	-0.060***
	(0.0125)	(0.0112)	(0.0112)	(0.0125)	(0.0112)	(0.0112)
Years education	-0.022***	-0.006***	-0.008***	-0.022***	-0.008***	-0.008***
	(0.0012)	(0.0011)	(0.0011)	(0.0012)	(0.0011)	(0.0011)
In paid work	-0.260***	-0.194***	-0.187***	-0.261***	-0.188***	-0.188***

	(0.0102)	(0.0092)	(0.0091)	(0.0102)	(0.0091)	(0.0091)
Belgium	-0.832***	-0.242***	-0.249***	-0.835***	-0.248***	-0.251***
	(0.0261)	(0.0235)	(0.0234)	(0.0261)	(0.0234)	(0.0234)
Switzerland	0.037	0.437***	0.355***	0.039	0.356***	0.357***
	(0.0265)	(0.0239)	(0.0240)	(0.0265)	(0.0240)	(0.0240)
Germany	-0.675***	-0.220***	-0.225***	-0.678***	-0.225***	-0.225***
	(0.0239)	(0.0219)	(0.0219)	(0.0239)	(0.0218)	(0.0219)
Denmark	-1.206***	-0.733***	-0.719***	-1.208***	-0.727***	-0.716***
	(0.0278)	(0.0261)	(0.0261)	(0.0278)	(0.0261)	(0.0261)
Spain	-0.207***	-0.163***	-0.155***	-0.209***	-0.154***	-0.158***
	(0.0260)	(0.0232)	(0.0231)	(0.0260)	(0.0231)	(0.0231)
Finland	-0.257***	0.213***	0.247***	-0.257***	0.238***	0.246***
	(0.0269)	(0.0254)	(0.0254)	(0.0269)	(0.0254)	(0.0254)
France	-1.061***	-0.585***	-0.589***	-1.064***	-0.588***	-0.587***
	(0.0271)	(0.0243)	(0.0243)	(0.0271)	(0.0243)	(0.0243)
UK	-0.650***	-0.004	-0.017	-0.654***	-0.017	-0.021
	(0.0264)	(0.0243)	(0.0243)	(0.0264)	(0.0243)	(0.0243)
Ireland	1.335***	1.209***	1.198***	1.330***	1.195***	1.195***
	(0.0250)	(0.0224)	(0.0223)	(0.0250)	(0.0223)	(0.0223)
Italy	0.574***	0.414***	0.439***	0.573***	0.440***	0.446***
	(0.0309)	(0.0276)	(0.0275)	(0.0309)	(0.0275)	(0.0276)
Netherlands	-0.608***	0.129***	0.132***	-0.610***	0.133***	0.132***
	(0.0258)	(0.0236)	(0.0235)	(0.0258)	(0.0235)	(0.0235)
Norway	-0.918***	-0.351***	-0.350***	-0.922***	-0.356***	-0.348***
	(0.0266)	(0.0249)	(0.0249)	(0.0266)	(0.0249)	(0.0249)
Portugal	0.689***	0.524***	0.535***	0.684***	0.535***	0.526***
	(0.0270)	(0.0242)	(0.0241)	(0.0270)	(0.0241)	(0.0242)
Sweden	-1.362***	-0.389***	-0.411***	-1.362***	-0.412***	-0.410***
	(0.0261)	(0.0243)	(0.0243)	(0.0261)	(0.0242)	(0.0243)
Observations	226,301	226,301	226,301	226,301	226,301	226,301
R-squared	0.216	0.375	0.379	0.217	0.378	0.379

Standard errors in parentheses
*** p<0.001, ** p<0.01, * p<0.05, + p<0.1
Notes: Model 1 includes generation but not religion, model 2 includes religion but not generation; Mod3l 3 includes religion and generation combined Model 4 includes generation X time trend, Model 5 includes generation X time trend controlling for religion, and Model 6 has religion and generation combined time trend

Table S5: Full estimates from OLS models of frequency of attendance

	Model 1	Model 2	Model 3	Model 4	Model 5	Model 6
ESS round	-0.028***	-0.018***	-0.019***	-0.033***	-0.022***	-0.014***
	(0.0011)	(0.0009)	(0.0009)	(0.0012)	(0.0010)	(0.0016)
1st generation	0.318***			0.119***	-0.011	
	(0.0097)			(0.0227)	(0.0201)	
2nd generation	0.085***			-0.066**	-0.055*	
	(0.0111)			(0.0249)	(0.0219)	
1st gen#ESS round				0.036***	0.025***	
				(0.0037)	(0.0033)	
2nd gen#ESS round				0.029***	0.013***	
				(0.0042)	(0.0037)	
Native Catholic			1.463***			1.604***
			(0.0072)			(0.0136)
Native protestant			1.045***			0.990***
			(0.0075)			(0.0152)
Native Oth Christian			2.155***			2.189***
			(0.0249)			(0.0514)
Native other religions			0.943***			0.886***
			(0.0397)			(0.0851)
1st Gen no religion			0.069***			0.037
			(0.0139)			(0.0323)
1st Gen Catholic			1.556***			1.511***
			(0.0162)			(0.0379)
1st Gen Protestant			1.574***			1.279***
			(0.0253)			(0.0585)
1st Gen Other Christian			2.119***			2.156***
			(0.0445)			(0.1079)
1st Gen Orthodox			1.338***			1.054***
			(0.0354)			(0.0924)
1st Gen Islam			1.671***			1.377***
			(0.0207)			(0.0519)
1st Gen Other			1.387***			1.336***
			(0.0414)			(0.1042)
2nd Gen no religion			0.023+			0.030
			(0.0141)			(0.0321)
2nd Gen Catholic			1.314***			1.412***
			(0.0185)			(0.0406)
2nd Gen Protestant			1.156***			0.936***
			(0.0256)			(0.0556)
2nd Gen Other Christian			2.243***			2.260***
			(0.0736)			(0.1582)

2nd Gen Orthodox	1.537***	1.224***
	(0.0948)	(0.2331)
2nd Gen Islam	1.832***	1.652***
	(0.0328)	(0.0900)
2nd Gen Other	1.385***	1.074***
	(0.0735)	(0.1754)
Nat Cath#ESS round		-0.028***
		(0.0023)
Nat Prot##ESS round		0.012***
		(0.0027)
Nat Oth Christian##ESS round		-0.007
		(0.0093)
Nat Oth##ESS round		0.011
		(0.0148)
1 Gen No rel#ESS round		0.005
		(0.0053)
1 Gen Cath#ESS round		0.008
		(0.0063)
1 Gen Protl#ESS round		0.056***
		(0.0100)
1 Gen Oth Chr#ESS round		-0.007
		(0.0177)
1 Gen Orthodox#ESS round		0.047**
		(0.0143)
1 Gen Islam#ESS round		0.051***
		(0.0083)
1 Gen Other#ESS round		0.009
		(0.0167)
2 Gen No rel#ESS round		-0.001
		(0.0055)
2 Gen Cath#ESS round		-0.019**
		(0.0069)
2 Gen Protl#ESS round		0.044***
		(0.0099)
2 Gen Oth Chr#ESS round		-0.003
		(0.0270)

2 Gen Orthodox#ESS round						0.052
						(0.0361)
2 Gen Islam#ESS round						0.029*
						(0.0140)
2 Gen Other#ESS round						0.055+
						(0.0281)
Catholic		1.451***			1.451***	
		(0.0066)			(0.0066)	
Protestant		1.073***			1.076***	
		(0.0071)			(0.0071)	
Orthodox		1.350***			1.240***	
		(0.0332)			(0.0340)	
Oth Christian		2.146***			2.127***	
		(0.0210)			(0.0210)	
Islam		1.705***			1.617***	
		(0.0177)			(0.0188)	
Other		1.176***			1.132***	
		(0.0269)			(0.0270)	
Age	0.010***	0.006***	0.006***	0.010***	0.006***	0.006***
	(0.0002)	(0.0002)	(0.0002)	(0.0002)	(0.0002)	(0.0002)
Women	0.271***	0.177***	0.176***	0.271***	0.177***	0.176***
	(0.0055)	(0.0049)	(0.0049)	(0.0055)	(0.0049)	(0.0049)
Divorced widowed	-0.267***	-0.154***	-0.155***	-0.266***	-0.155***	-0.153***
	(0.0077)	(0.0067)	(0.0067)	(0.0077)	(0.0067)	(0.0067)
Never married	-0.239***	-0.115***	-0.111***	-0.238***	-0.110***	-0.110***
	(0.0075)	(0.0066)	(0.0066)	(0.0075)	(0.0066)	(0.0066)
Years education	-0.012***	-0.003***	-0.003***	-0.011***	-0.003***	-0.003***
	(0.0007)	(0.0006)	(0.0006)	(0.0007)	(0.0006)	(0.0006)
In paid work	-0.134***	-0.094***	-0.091***	-0.134***	-0.093***	-0.092***
	(0.0062)	(0.0054)	(0.0054)	(0.0062)	(0.0054)	(0.0054)
Belgium	-0.686***	-0.290***	-0.291***	-0.688***	-0.291***	-0.293***
	(0.0157)	(0.0139)	(0.0139)	(0.0157)	(0.0139)	(0.0139)
Switzerland	-0.275***	-0.030*	-0.043**	-0.274***	-0.046**	-0.040**
	(0.0159)	(0.0142)	(0.0142)	(0.0159)	(0.0142)	(0.0142)
Germany	-0.495***	-0.168***	-0.167***	-0.497***	-0.169***	-0.165***
	(0.0144)	(0.0129)	(0.0129)	(0.0144)	(0.0129)	(0.0129)
Denmark	-0.553***	-0.184***	-0.172***	-0.554***	-0.183***	-0.167***
	(0.0167)	(0.0154)	(0.0155)	(0.0167)	(0.0154)	(0.0155)
Spain	-0.266***	-0.238***	-0.241***	-0.267***	-0.238***	-0.243***
	(0.0156)	(0.0137)	(0.0137)	(0.0156)	(0.0137)	(0.0137)
Finland	-0.427***	-0.054***	-0.034*	-0.426***	-0.049**	-0.036*
	(0.0162)	(0.0150)	(0.0151)	(0.0162)	(0.0150)	(0.0151)
France	-0.727***	-0.409***	-0.408***	-0.729***	-0.409***	-0.402***
	(0.0163)	(0.0144)	(0.0144)	(0.0163)	(0.0144)	(0.0144)
UK	-0.589***	-0.128***	-0.127***	-0.590***	-0.131***	-0.131***
	(0.0158)	(0.0144)	(0.0144)	(0.0158)	(0.0144)	(0.0144)
Ireland	0.845***	0.746***	0.739***	0.843***	0.741***	0.738***
	(0.0150)	(0.0132)	(0.0132)	(0.0150)	(0.0132)	(0.0132)

Italy	0.444***	0.344***	0.343***	0.443***	0.347***	0.355***
	(0.0186)	(0.0163)	(0.0163)	(0.0185)	(0.0163)	(0.0163)
Netherlands	-0.657***	-0.145***	-0.141***	-0.658***	-0.144***	-0.141***
	(0.0155)	(0.0139)	(0.0139)	(0.0155)	(0.0139)	(0.0139)
Norway	-0.516***	-0.095***	-0.087***	-0.518***	-0.097***	-0.083***
	(0.0160)	(0.0147)	(0.0148)	(0.0160)	(0.0147)	(0.0148)
Portugal	0.222***	0.106***	0.101***	0.220***	0.107***	0.091***
	(0.0162)	(0.0143)	(0.0143)	(0.0162)	(0.0143)	(0.0143)
Sweden	-0.647***	0.013	0.013	-0.647***	0.009	0.016
	(0.0157)	(0.0144)	(0.0144)	(0.0157)	(0.0144)	(0.0144)
Observations	226,301	226,301	226,301	226,301	226,301	226,301
R-squared	0.167	0.357	0.359	0.168	0.358	0.360

Standard errors in parentheses
*** p<0.001, ** p<0.01, * p<0.05, + p<0.1
Notes: Model 1 includes generation but not religion, model 2 includes religion but not generation; Mod3l 3 includes religion and generation combined Model 4 includes generation X time trend, Model 5 includes generation X time trend controlling for religion, and Model 6 has religion and generation combined time trend

Table S6: Full estimates from OLS models of frequency of subjective religiosity

	Model 1	Model 2	Model 3	Model 4	Model 5	Model 6
ESS round	-0.072***	-0.049***	-0.052***	-0.083***	-0.057***	-0.097***
	(0.0023)	(0.0020)	(0.0020)	(0.0025)	(0.0021)	(0.0033)
1st generation	1.170***			0.773***	0.391***	
	(0.0207)			(0.0485)	(0.0419)	
2nd generation	0.439***			0.071	0.083+	
	(0.0238)			(0.0533)	(0.0456)	
1st gen#ESS round				0.073***	0.038***	
				(0.0080)	(0.0068)	
2nd gen#ESS round				0.070***	0.027***	
				(0.0091)	(0.0078)	
Native Catholic			3.174***			2.842***
			(0.0149)			(0.0283)
Native protestant			2.669***			2.286***
			(0.0157)			(0.0316)
Native Oth Christian			4.576***			4.232***
			(0.0519)			(0.1071)
Native other religions			3.538***			3.502***
			(0.0826)			(0.1771)
1st Gen no religion			0.601***			0.604***
			(0.0289)			(0.0672)
1st Gen Catholic			3.784***			3.229***
			(0.0337)			(0.0789)
1st Gen Protestant			3.585***			2.695***
			(0.0528)			(0.1217)
1st Gen Other Christian			4.960***			3.963***
			(0.0927)			(0.2247)
1st Gen Orthodox			3.930***			3.503***
			(0.0737)			(0.1924)
1st Gen Islam			4.728***			3.899***
			(0.0431)			(0.1080)
1st Gen Other			4.233***			3.502***
			(0.0862)			(0.2170)
2nd Gen no religion			0.178***			0.157*
			(0.0293)			(0.0668)
2nd Gen Catholic			3.261***			2.790***
			(0.0386)			(0.0845)
2nd Gen Protestant			2.964***			2.060***
			(0.0534)			(0.1159)
2nd Gen Other Christian			4.826***			4.684***
			(0.1532)			(0.3295)
2nd Gen Orthodox			4.029***			3.869***
			(0.1974)			(0.4854)
2nd Gen Islam			5.076***			4.488***
			(0.0683)			(0.1874)
2nd Gen Other			3.878***			3.259***
			(0.1530)			(0.3651)
Nat Cath#ESS round						0.065***

			(0.0048)
Nat Prot##ESS round			0.076***
			(0.0056)
Nat Oth Christian##ESS round			0.068***
			(0.0193)
Nat Oth##ESS round			0.007
			(0.0309)
1 Gen No rel#ESS round			0.002
			(0.0111)
1 Gen Cath#ESS round			0.104***
			(0.0132)
1 Gen Protl#ESS round			0.170***
			(0.0208)
1 Gen Oth Chr#ESS round			0.182***
			(0.0369)
1 Gen Orthodox#ESS round			0.078**
			(0.0298)
1 Gen Islam#ESS round			0.148***
			(0.0172)
1 Gen Other#ESS round			0.132***
			(0.0348)
2 Gen No rel#ESS round			0.005
			(0.0114)
2 Gen Cath#ESS round			0.090***
			(0.0145)
2 Gen Protl#ESS round			0.180***
			(0.0206)
2 Gen Oth Chr#ESS round			0.028
			(0.0561)
2 Gen Orthodox#ESS round			0.033
			(0.0751)
2 Gen Islam#ESS round			0.104***
			(0.0291)
2 Gen Other#ESS round			0.113+
			(0.0585)
Catholic	3.169***	3.169***	
	(0.0138)	(0.0138)	
Protestant	2.672***	2.692***	

		(0.0148)			(0.0148)	
Orthodox		3.866***			3.374***	
		(0.0693)			(0.0708)	
Oth Christian		4.620***			4.533***	
		(0.0438)			(0.0438)	
Islam		4.749***			4.321***	
		(0.0369)			(0.0392)	
Other		3.801***			3.591***	
		(0.0560)			(0.0563)	
Age	0.028***	0.018***	0.019***	0.028***	0.019***	0.019***
	(0.0004)	(0.0004)	(0.0004)	(0.0004)	(0.0004)	(0.0004)
Women	0.873***	0.663***	0.658***	0.873***	0.659***	0.658***
	(0.0118)	(0.0102)	(0.0102)	(0.0118)	(0.0102)	(0.0101)
Divorced widowed	-0.324***	-0.062***	-0.068***	-0.323***	-0.066***	-0.070***
	(0.0164)	(0.0141)	(0.0140)	(0.0163)	(0.0140)	(0.0140)
Never married	-0.480***	-0.195***	-0.176***	-0.479***	-0.172***	-0.175***
	(0.0161)	(0.0138)	(0.0138)	(0.0161)	(0.0138)	(0.0138)
Years education	-0.034***	-0.012***	-0.014***	-0.034***	-0.014***	-0.014***
	(0.0015)	(0.0013)	(0.0013)	(0.0015)	(0.0013)	(0.0013)
In paid work	-0.196***	-0.099***	-0.090***	-0.197***	-0.093***	-0.091***
	(0.0131)	(0.0113)	(0.0113)	(0.0131)	(0.0113)	(0.0112)
Belgium	-0.236***	0.633***	0.627***	-0.240***	0.628***	0.627***
	(0.0335)	(0.0289)	(0.0289)	(0.0335)	(0.0289)	(0.0289)
Switzerland	-0.053	0.465***	0.378***	-0.050	0.377***	0.388***
	(0.0340)	(0.0295)	(0.0296)	(0.0340)	(0.0296)	(0.0296)
Germany	-0.952***	-0.313***	-0.316***	-0.955***	-0.317***	-0.318***
	(0.0307)	(0.0270)	(0.0270)	(0.0307)	(0.0269)	(0.0269)
Denmark	-0.748***	-0.118***	-0.101**	-0.750***	-0.109***	-0.099**
	(0.0356)	(0.0322)	(0.0322)	(0.0356)	(0.0321)	(0.0322)
Spain	-0.539***	-0.469***	-0.457***	-0.541***	-0.456***	-0.448***
	(0.0333)	(0.0285)	(0.0285)	(0.0333)	(0.0285)	(0.0285)
Finland	0.258***	0.887***	0.927***	0.258***	0.917***	0.937***
	(0.0345)	(0.0313)	(0.0314)	(0.0345)	(0.0313)	(0.0314)
France	-0.753***	-0.049	-0.053+	-0.757***	-0.053+	-0.046
	(0.0348)	(0.0300)	(0.0299)	(0.0348)	(0.0299)	(0.0299)
UK	-1.016***	-0.102***	-0.112***	-1.020***	-0.115***	-0.109***
	(0.0338)	(0.0300)	(0.0300)	(0.0338)	(0.0299)	(0.0300)
Ireland	0.395***	0.205***	0.194***	0.390***	0.193***	0.201***
	(0.0321)	(0.0276)	(0.0275)	(0.0321)	(0.0275)	(0.0275)
Italy	0.914***	0.691***	0.721***	0.913***	0.722***	0.714***
	(0.0396)	(0.0340)	(0.0340)	(0.0396)	(0.0339)	(0.0340)
Netherlands	-0.328***	0.750***	0.757***	-0.330***	0.756***	0.760***
	(0.0331)	(0.0290)	(0.0290)	(0.0331)	(0.0290)	(0.0290)
Norway	-1.002***	-0.229***	-0.224***	-1.006***	-0.232***	-0.226***
	(0.0341)	(0.0307)	(0.0307)	(0.0341)	(0.0307)	(0.0307)
Portugal	0.313***	0.085**	0.097**	0.308***	0.100***	0.111***
	(0.0347)	(0.0298)	(0.0298)	(0.0347)	(0.0297)	(0.0298)
Sweden	-1.574***	-0.189***	-0.209***	-1.574***	-0.212***	-0.222***
	(0.0335)	(0.0300)	(0.0299)	(0.0335)	(0.0299)	(0.0299)
Observations	226,301	226,301	226,301	226,301	226,301	226,301
R-squared	0.141	0.368	0.371	0.141	0.371	0.373

Standard errors in parentheses. *** p<0.001, ** p<0.01, * p<0.05, + p<0.1.

Notes: Model 1 includes generation but not religion, model 2 includes religion but not generation; Mod3l 3 includes religion and generation combined Model 4 includes generation X time trend, Model 5 includes generation X time trend controlling for religion, and Model 6 has religion X generation combined time trend.

FIGURES

Figure S1: Trends in religiosity by migrant status and by religious affiliation and migrant status: using nominal measure for ESS round

A. Prayer: Native no religion and Catholic

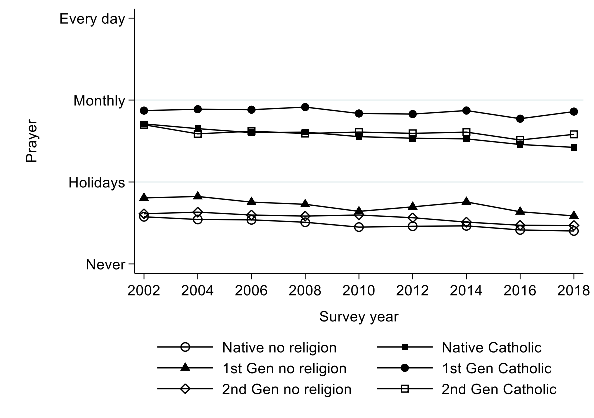

B. Prayer: Native no religion, Protestant, Muslim

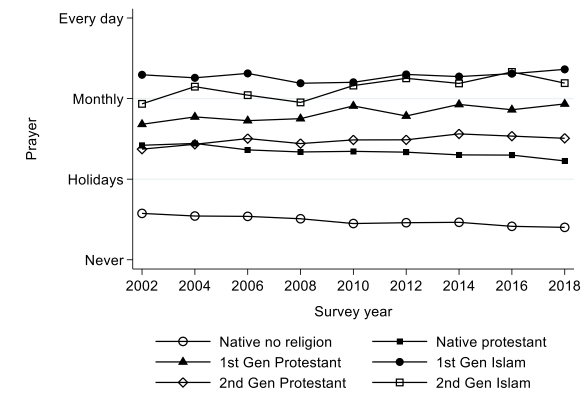

C. Prayer: Native no religion, Other Christian, Orthodox

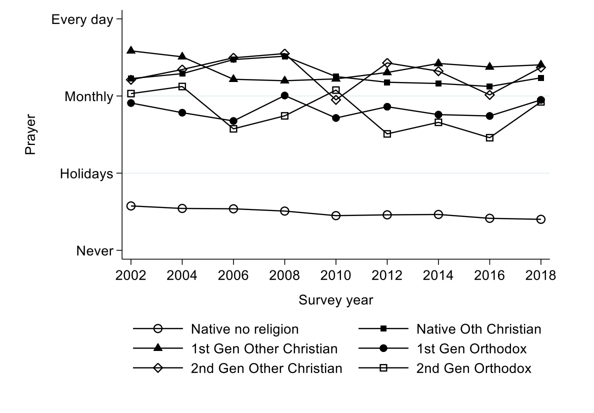

D. Attendance: Native no religion and Catholic

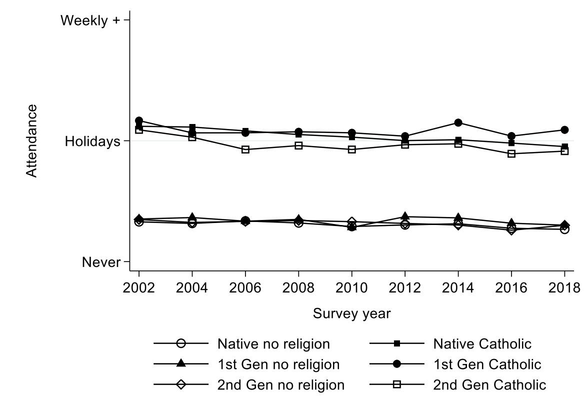

E. Attendance: Native no religion, Protestant, Muslim

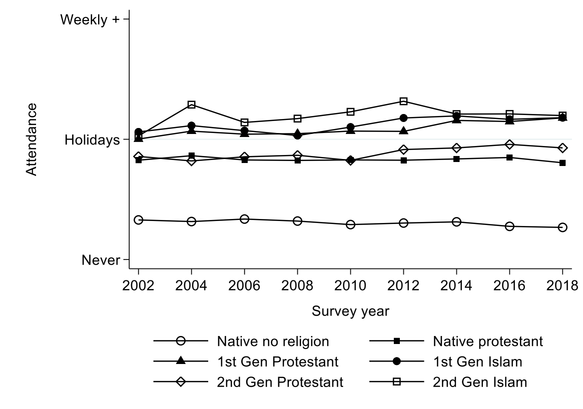

F. Attendance: Native no religion, Other Christian, Orthodox

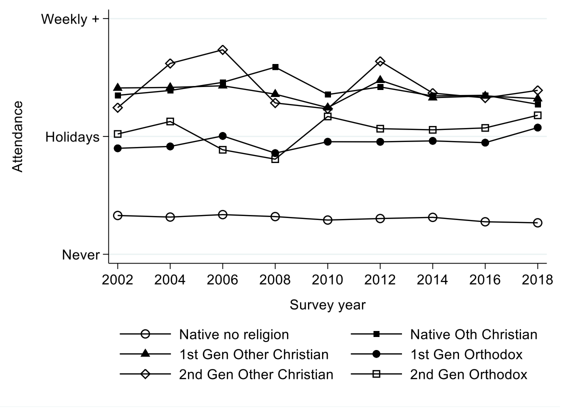

G. Subjective religiosity: Native no religion and Catholic

H. Subjective religiosity: Native no religion, Protestant, Muslim

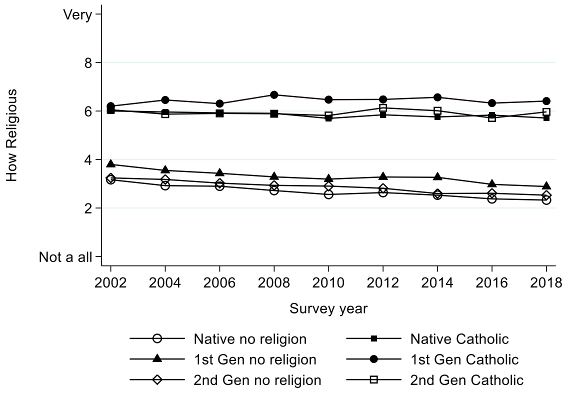

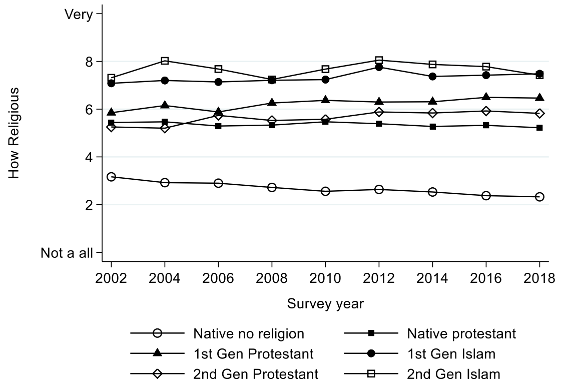

I. Subjective religiosity: Native no religion, Other Christian, Orthodox

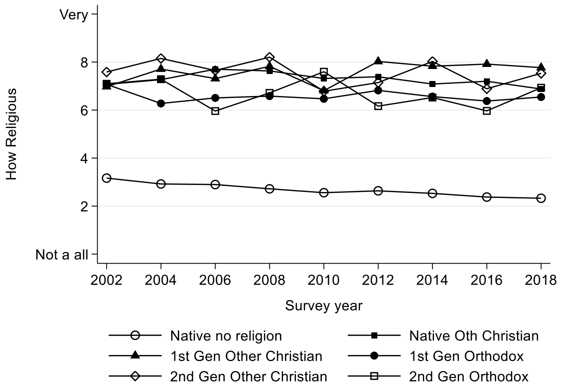

Source: ESS Rounds 1(2002) – 9 (2018); estimates control for age, sex, marital status, educational level, and country fixed effects.
